# Benzyl 5-phenyl­pyrazolo­[5,1-*a*]isoquino­line-1-carboxyl­ate

**DOI:** 10.1107/S1600536811050586

**Published:** 2011-11-30

**Authors:** Yu-Kun Lu, Xiao Yao, Li-Wen Luo, Ren-Qing Lü, Yun-Qi Liu

**Affiliations:** a State Key Laboratory of Heavy Oil Processing, College of Science, China University of Petroleum (East China), Qingdao Shandong 266555, People’s Republic of China; b State Key Laboratory of Heavy Oil Processing, College of Chemical Engineering, China University of Petroleum (East China), Qingdao Shandong 266555, People’s Republic of China

## Abstract

In the title compound, C_25_H_18_N_2_O_2_, the pyrazolo­[5,1-*a*]iso­quin­oline ring system is approximately planar [maximum deviation = 0.027 (2) Å] and is oriented at dihedral angles of 57.22 (6) and 71.36 (7)° with respect to the two phenyl rings. The phenyl rings are twisted to each other by a dihedral angle of 66.33 (8)°. A weak intra­molecular C—H⋯O hydrogen bond occurs. In the crystal, weak C—H⋯π inter­actions are present.

## Related literature

For the biological activity of fused isoquinoline compounds, see: Aubry *et al.* (2004 ▶); Marco *et al.* (2005 ▶); Reddy *et al.* (1999 ▶). For related structures, see: Chen & Wu (2010 ▶); Ye *et al.* (2010 ▶); Yu *et al.* (2011*a* ▶,*b* ▶). For selected examples of multi-component reactions, see: Dömling & Ugi (2000 ▶); Nair *et al.* (2003 ▶); Ramon & Yus (2005 ▶).
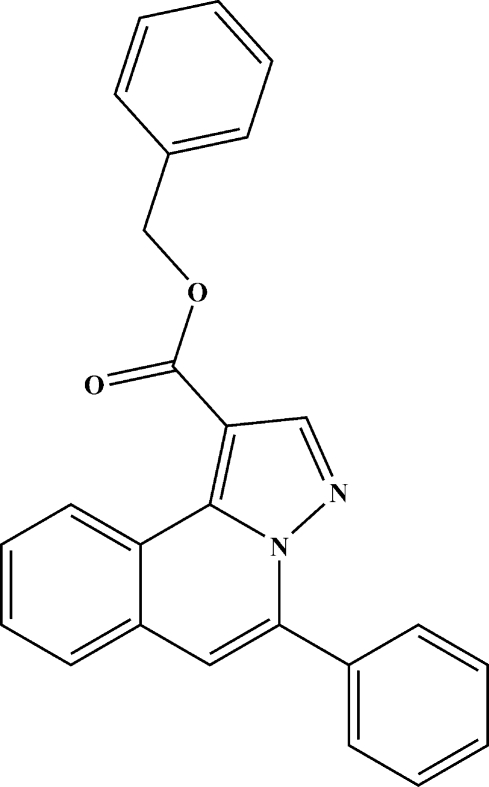


## Experimental

### 

#### Crystal data

C_25_H_18_N_2_O_2_
*M* *_r_* = 378.41
Monoclinic, 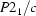

*a* = 9.161 (3) Å
*b* = 18.397 (6) Å
*c* = 11.621 (4) Å
β = 98.132 (6)°
*V* = 1938.9 (12) Å^3^
*Z* = 4
Mo *K*α radiation
μ = 0.08 mm^−1^
*T* = 173 K
0.22 × 0.15 × 0.11 mm

#### Data collection

Rigaku R-AXIS RAPID diffractometer
9583 measured reflections
3378 independent reflections
1852 reflections with *I* > 2σ(*I*)
*R*_int_ = 0.098

#### Refinement

*R*[*F* ^2^ > 2σ(*F* ^2^)] = 0.050
*wR*(*F* ^2^) = 0.116
*S* = 0.87
3378 reflections
263 parameters
H-atom parameters constrained
Δρ_max_ = 0.18 e Å^−3^
Δρ_min_ = −0.24 e Å^−3^

### 

Data collection: *PROCESS-AUTO* (Rigaku, 1998 ▶); cell refinement: *PROCESS-AUTO*; data reduction: *PROCESS-AUTO*; program(s) used to solve structure: *SHELXS97* (Sheldrick, 2008 ▶); program(s) used to refine structure: *SHELXL97* (Sheldrick, 2008 ▶); molecular graphics: *DIAMOND* (Brandenburg, 1999 ▶); software used to prepare material for publication: *SHELXL97*.

## Supplementary Material

Crystal structure: contains datablock(s) I, New_Global_Publ_Block. DOI: 10.1107/S1600536811050586/xu5395sup1.cif

Structure factors: contains datablock(s) I. DOI: 10.1107/S1600536811050586/xu5395Isup2.hkl

Additional supplementary materials:  crystallographic information; 3D view; checkCIF report

## Figures and Tables

**Table 1:** Hydrogen-bond geometry (Å, °) *Cg*1 and *Cg*2 are the centroids of the N1/N2C11/C16/C17 and C20–C25 rings, respectively.

*D*—H⋯*A*	*D*—H	H⋯*A*	*D*⋯*A*	*D*—H⋯*A*
C15—H15*A*⋯O1	0.95	2.17	3.012 (3)	148
C1—H1*A*⋯*Cg*1^i^	0.95	2.76	3.484 (3)	134
C14—H14*A*⋯*Cg*2^ii^	0.95	2.68	3.594 (3)	161

Symmetry codes: (i) 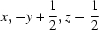; (ii) 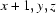.

**Fig. 1.**
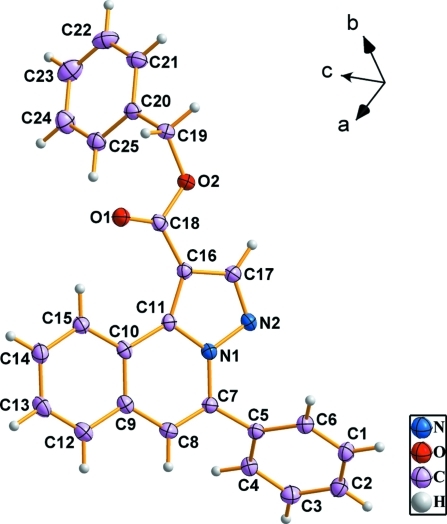
The molecular structure of the title compound, with 30% probability displacement ellipsoids and the atom-numbering scheme.

**Fig. 2.**
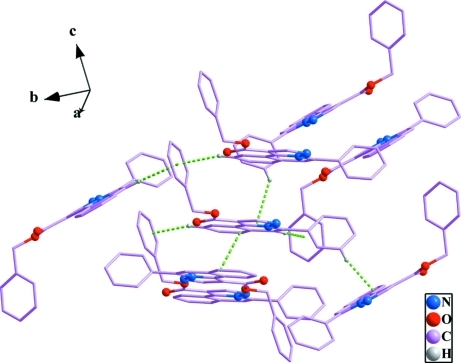
The arrangement of the molecules in the crystal structure. The π-π stacking and C—H···π interactions are represented by dashed lines. H atoms not involved in interactions have been omitted for clarity.

**Fig. 3.**
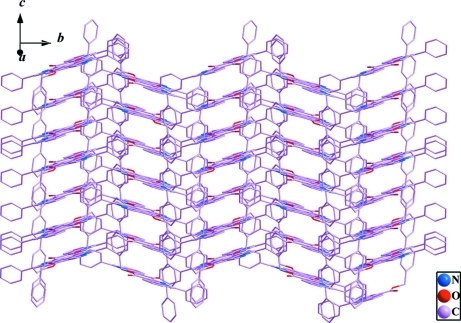
The crystal packing of the title compound viewed along the [201] direction.

## supplementary crystallographic information

### Comment

In the last decade, diversity-oriented synthesis has been widely used to efficiently generate diverse small molecules. Among the strategies employed in diversity-oriented chemical synthesis, multi-component reactions are very attractive processes that push the limits of synthetic efficiency by using more than two reactants to create novel products with an optimal number of new bonds and functionalities (Dömling & Ugi, 2000; Nair *et al.*, 2003; Ramon & Yus, 2005). Among the family of isoquinolines, the fused isoquinolines have attracted much attention owing to their biological activities including potent inhibitor of human topoisomerase I and selective inhibition against HIV-1 integrase *in vitro* (Aubry *et al.*, 2004; Marco *et al.*, 2005; Reddy *et al.*, 1999). We report herein on the single-crystal X-ray diffraction study of the title compound, synthesized from 2-(phenylethynyl)benzaldehyde, sulfonohydrazide and benzyl acrylate in DCE/DMAc.

The molecular structure of the title compound is shown in Fig. 1, the bond lengths and angles are normal and correspond to those observed in related structures (Chen & Wu, 2010; Ye *et al.*, 2010; Yu *et al.*, 2011*a*; Yu *et al.*, 2011*b*). It is compound of three aromatic rings namely, a pyrazolo[5,1-*a*]isoquinoline ring [A = (N1, N2, C7—C17)], two benzene ring [B = (C1—C6)] and [C = (C20—C25], with the dihedral angles of 57.22 (6)°, 71.36 (6)° and 66.33 (8)° between the mean planes A/B, A/C and B/C, respectively; and the carboxyl group is twisted at an angle of 8.78 (9)° relative to the A skeleton. Atoms C16 in A ring and C20 in the benzene ring are joined by the ester group (C18/O1/O2/C19) giving the torsion angles C18—O2—C19—C20 and C19—O2—C18—C16 are -96.2 (2)° and -179.81 (17)°, respectively. Atom N1 has a trigonal configuration, the sum of three bond angles around it being 360°. The mean planes of the adjacent A moieties are parallel [at an angle 0.00 (5)°] or inclined at an angle of 37.69 (4)° in the crystal lattice.

In the crystal structure, molecules are connected *via* C—H···π interactions (Fig. 2 and Table 1), forming a three-dimensional supramolecular framework (Fig. 3), where *Cg*1 and *Cg*2 are the centroids of C11, C16, C17, N1, N2 and C20–C25 rings, respectively.

### Experimental

The reaction was performed in test tube under nitrogen atmosphere. 2-(phenylethynyl)benzaldehyde (0.2 mmol) was added to a solution of sulfonohydrazide (0.2 mmol) in DCE (0.5 ml). The mixture was stirred at room temperature for 30 min. Then AgOTf (7.7 mg, 0.01 mmol) was added and the reaction mixture was heated to 70 *^o^*C for 1 h. Subsequently, benzyl acrylate (0.4 mmol) and DMAc (2 ml) were added in the mixture. After completion of reaction as indicated by TLC, the reaction was quenched with aqueous NH_4_Cl (10 ml, 1.0 *M*), extracted with EtOAc (10 ml), dried by anhydrous Na_2_SO_4_. Evaporation of the solvent followed by purification on silica gel provided the crystals suitable for X-ray analysis.

### Refinement

H atoms were positioned geometrically with C—H = 0.95–0.99 Å, and constrained to ride on their parent atoms with *U*_iso_(H) = 1.2*U*_eq_(C).

### Crystal data

**Table:**

C_25_H_18_N_2_O_2_	*F*(000) = 792
*M**_r_* = 378.41	*D*_x_ = 1.296 Mg m^−^^3^
Monoclinic, *P*2_1_/*c*	Mo *K*α radiation, λ = 0.71073 Å
Hall symbol: -P 2ybc	Cell parameters from 3378 reflections
*a* = 9.161 (3) Å	θ = 2.1–25.0°
*b* = 18.397 (6) Å	µ = 0.08 mm^−^^1^
*c* = 11.621 (4) Å	*T* = 173 K
β = 98.132 (6)°	Block, colorless
*V* = 1938.9 (12) Å^3^	0.22 × 0.15 × 0.11 mm
*Z* = 4	

### Data collection

**Table:**

Rigaku R-AXIS RAPID diffractometer	1852 reflections with *I* > 2σ(*I*)
Radiation source: fine-focus sealed tube	*R*_int_ = 0.098
graphite	θ_max_ = 25.0°, θ_min_ = 2.1°
Detector resolution: 10 pixels mm^-1^	*h* = −9→10
ω scans	*k* = −20→21
9583 measured reflections	*l* = −13→13
3378 independent reflections	

### Refinement

**Table:**

Refinement on *F*^2^	Secondary atom site location: difference Fourier map
Least-squares matrix: full	Hydrogen site location: inferred from neighbouring sites
*R*[*F*^2^ > 2σ(*F*^2^)] = 0.050	H-atom parameters constrained
*wR*(*F*^2^) = 0.116	*w* = 1/[σ^2^(*F*_o_^2^) + (0.*P*)^2^] where *P* = (*F*_o_^2^ + 2*F*_c_^2^)/3
*S* = 0.87	(Δ/σ)_max_ < 0.001
3378 reflections	Δρ_max_ = 0.18 e Å^−^^3^
263 parameters	Δρ_min_ = −0.24 e Å^−^^3^
0 restraints	Extinction correction: *SHELXL*, Fc^*^=kFc[1+0.001xFc^2^λ^3^/sin(2θ)]^-1/4^
Primary atom site location: structure-invariant direct methods	Extinction coefficient: 0.0076 (11)

### Special details

**Table:**

Geometry. All e.s.d.'s (except the e.s.d. in the dihedral angle between two l.s. planes) are estimated using the full covariance matrix. The cell e.s.d.'s are taken into account individually in the estimation of e.s.d.'s in distances, angles and torsion angles; correlations between e.s.d.'s in cell parameters are only used when they are defined by crystal symmetry. An approximate (isotropic) treatment of cell e.s.d.'s is used for estimating e.s.d.'s involving l.s. planes.
Refinement. Refinement of *F*^2^ against ALL reflections. The weighted *R*-factor *wR* and goodness of fit *S* are based on *F*^2^, conventional *R*-factors *R* are based on *F*, with *F* set to zero for negative *F*^2^. The threshold expression of *F*^2^ > σ(*F*^2^) is used only for calculating *R*-factors(gt) *etc*. and is not relevant to the choice of reflections for refinement. *R*-factors based on *F*^2^ are statistically about twice as large as those based on *F*, and *R*- factors based on ALL data will be even larger.

### Fractional atomic coordinates and isotropic or equivalent isotropic displacement parameters (Å^2^)

**Table:**

	*x*	*y*	*z*	*U*_iso_*/*U*_eq_	
N1	0.4210 (2)	0.36318 (10)	0.06266 (15)	0.0314 (5)	
N2	0.2711 (2)	0.36122 (10)	0.01947 (16)	0.0355 (5)	
O1	0.41215 (19)	0.59556 (8)	0.15872 (15)	0.0450 (5)	
O2	0.17077 (19)	0.56806 (8)	0.10441 (14)	0.0401 (5)	
C1	0.3320 (3)	0.16526 (14)	−0.1618 (2)	0.0455 (7)	
H1A	0.2871	0.1641	−0.2407	0.055*	
C2	0.3482 (3)	0.10140 (14)	−0.0978 (2)	0.0486 (7)	
H2B	0.3152	0.0567	−0.1332	0.058*	
C3	0.4124 (3)	0.10268 (13)	0.0176 (2)	0.0476 (7)	
H3A	0.4236	0.0589	0.0612	0.057*	
C4	0.4604 (3)	0.16836 (12)	0.0697 (2)	0.0405 (7)	
H4A	0.5029	0.1693	0.1491	0.049*	
C5	0.4462 (3)	0.23300 (12)	0.0054 (2)	0.0339 (6)	
C6	0.3809 (3)	0.23078 (13)	−0.1113 (2)	0.0414 (7)	
H6A	0.3701	0.2743	−0.1557	0.050*	
C7	0.5104 (3)	0.30141 (12)	0.06079 (19)	0.0335 (6)	
C8	0.6539 (3)	0.30793 (12)	0.1091 (2)	0.0387 (6)	
H8A	0.7168	0.2669	0.1095	0.046*	
C9	0.7137 (3)	0.37483 (13)	0.1595 (2)	0.0359 (6)	
C10	0.6211 (3)	0.43691 (12)	0.16259 (19)	0.0320 (6)	
C11	0.4672 (3)	0.43004 (12)	0.11022 (19)	0.0298 (6)	
C12	0.8650 (3)	0.38081 (14)	0.2056 (2)	0.0468 (7)	
H12A	0.9282	0.3402	0.2021	0.056*	
C13	0.9217 (3)	0.44468 (14)	0.2553 (2)	0.0496 (7)	
H13A	1.0236	0.4480	0.2851	0.059*	
C14	0.8296 (3)	0.50468 (14)	0.2621 (2)	0.0463 (7)	
H14A	0.8684	0.5480	0.2992	0.056*	
C15	0.6817 (3)	0.50136 (12)	0.2150 (2)	0.0384 (7)	
H15A	0.6208	0.5429	0.2180	0.046*	
C16	0.3387 (3)	0.47398 (12)	0.09507 (19)	0.0308 (6)	
C17	0.2256 (3)	0.42822 (12)	0.0405 (2)	0.0363 (6)	
H17A	0.1263	0.4437	0.0207	0.044*	
C18	0.3186 (3)	0.55095 (13)	0.1236 (2)	0.0352 (6)	
C19	0.1330 (3)	0.64296 (12)	0.1286 (2)	0.0403 (7)	
H19A	0.0503	0.6591	0.0700	0.048*	
H19B	0.2187	0.6747	0.1218	0.048*	
C20	0.0897 (3)	0.65106 (12)	0.2487 (2)	0.0366 (6)	
C21	−0.0315 (3)	0.69401 (12)	0.2649 (2)	0.0462 (7)	
H21A	−0.0878	0.7166	0.1998	0.055*	
C22	−0.0709 (3)	0.70417 (14)	0.3754 (3)	0.0585 (9)	
H22A	−0.1525	0.7343	0.3851	0.070*	
C23	0.0080 (4)	0.67062 (15)	0.4707 (3)	0.0622 (9)	
H23A	−0.0197	0.6773	0.5457	0.075*	
C24	0.1283 (3)	0.62686 (15)	0.4565 (2)	0.0548 (8)	
H24A	0.1831	0.6037	0.5219	0.066*	
C25	0.1679 (3)	0.61719 (13)	0.3465 (2)	0.0434 (7)	
H25A	0.2496	0.5870	0.3374	0.052*	

### Atomic displacement parameters (Å^2^)

**Table:**

	*U*^11^	*U*^22^	*U*^33^	*U*^12^	*U*^13^	*U*^23^
N1	0.0286 (13)	0.0336 (11)	0.0314 (12)	−0.0006 (10)	0.0014 (9)	−0.0017 (9)
N2	0.0279 (13)	0.0405 (12)	0.0365 (12)	0.0008 (11)	−0.0010 (9)	0.0006 (9)
O1	0.0367 (12)	0.0391 (10)	0.0573 (12)	−0.0019 (9)	0.0002 (9)	−0.0078 (8)
O2	0.0341 (11)	0.0390 (10)	0.0454 (11)	0.0070 (9)	−0.0014 (8)	−0.0059 (8)
C1	0.0453 (19)	0.0571 (17)	0.0327 (16)	0.0032 (15)	0.0011 (13)	−0.0113 (13)
C2	0.0451 (19)	0.0430 (16)	0.056 (2)	−0.0034 (14)	0.0031 (15)	−0.0117 (14)
C3	0.050 (2)	0.0409 (16)	0.0511 (19)	0.0027 (14)	0.0032 (15)	0.0019 (13)
C4	0.0387 (17)	0.0444 (15)	0.0364 (15)	0.0014 (14)	−0.0012 (12)	−0.0027 (12)
C5	0.0309 (16)	0.0379 (14)	0.0327 (15)	0.0050 (12)	0.0039 (11)	−0.0019 (11)
C6	0.0427 (18)	0.0430 (15)	0.0372 (16)	0.0037 (13)	0.0016 (12)	−0.0028 (12)
C7	0.0348 (17)	0.0340 (14)	0.0313 (15)	0.0058 (13)	0.0030 (12)	−0.0011 (11)
C8	0.0380 (17)	0.0358 (14)	0.0408 (16)	0.0084 (14)	0.0005 (12)	0.0008 (11)
C9	0.0320 (16)	0.0419 (15)	0.0326 (15)	0.0000 (13)	0.0004 (12)	0.0023 (11)
C10	0.0298 (16)	0.0376 (14)	0.0282 (14)	−0.0023 (13)	0.0031 (11)	0.0054 (11)
C11	0.0310 (16)	0.0323 (13)	0.0259 (13)	−0.0017 (12)	0.0033 (11)	0.0011 (10)
C12	0.0360 (18)	0.0512 (17)	0.0509 (18)	0.0050 (15)	−0.0018 (13)	0.0007 (13)
C13	0.0307 (17)	0.0597 (18)	0.0552 (19)	−0.0032 (16)	−0.0046 (13)	−0.0001 (15)
C14	0.0427 (19)	0.0461 (16)	0.0466 (17)	−0.0047 (15)	−0.0061 (14)	−0.0030 (12)
C15	0.0354 (17)	0.0373 (15)	0.0407 (16)	−0.0003 (13)	−0.0004 (12)	0.0004 (12)
C16	0.0308 (16)	0.0344 (14)	0.0267 (14)	−0.0002 (13)	0.0021 (11)	−0.0014 (11)
C17	0.0344 (17)	0.0389 (15)	0.0348 (15)	0.0046 (13)	0.0023 (12)	−0.0019 (11)
C18	0.0343 (17)	0.0422 (16)	0.0284 (14)	0.0039 (14)	0.0026 (12)	0.0027 (11)
C19	0.0403 (17)	0.0348 (14)	0.0427 (16)	0.0098 (13)	−0.0045 (12)	−0.0019 (11)
C20	0.0291 (16)	0.0317 (14)	0.0479 (17)	−0.0012 (13)	0.0018 (12)	−0.0028 (12)
C21	0.0360 (18)	0.0400 (15)	0.063 (2)	0.0044 (14)	0.0078 (14)	0.0011 (13)
C22	0.052 (2)	0.0469 (17)	0.082 (3)	0.0066 (16)	0.0289 (18)	−0.0035 (16)
C23	0.070 (2)	0.0604 (19)	0.063 (2)	−0.0046 (19)	0.0314 (19)	−0.0062 (17)
C24	0.055 (2)	0.0675 (19)	0.0416 (18)	−0.0006 (17)	0.0048 (15)	0.0052 (14)
C25	0.0349 (17)	0.0518 (16)	0.0436 (17)	−0.0002 (14)	0.0065 (13)	−0.0037 (13)

### Geometric parameters (Å, °)

**Table:**

N1—C11	1.390 (3)	C11—C16	1.418 (3)
N1—N2	1.394 (3)	C12—C13	1.379 (3)
N1—C7	1.403 (3)	C12—H12A	0.9500
N2—C17	1.335 (3)	C13—C14	1.398 (3)
O1—C18	1.215 (3)	C13—H13A	0.9500
O2—C18	1.377 (3)	C14—C15	1.389 (3)
O2—C19	1.458 (2)	C14—H14A	0.9500
C1—C2	1.387 (3)	C15—H15A	0.9500
C1—C6	1.387 (3)	C16—C17	1.414 (3)
C1—H1A	0.9500	C16—C18	1.472 (3)
C2—C3	1.387 (4)	C17—H17A	0.9500
C2—H2B	0.9500	C19—C20	1.511 (3)
C3—C4	1.394 (3)	C19—H19A	0.9900
C3—H3A	0.9500	C19—H19B	0.9900
C4—C5	1.400 (3)	C20—C21	1.397 (3)
C4—H4A	0.9500	C20—C25	1.401 (3)
C5—C6	1.403 (3)	C21—C22	1.395 (4)
C5—C7	1.496 (3)	C21—H21A	0.9500
C6—H6A	0.9500	C22—C23	1.379 (4)
C7—C8	1.360 (3)	C22—H22A	0.9500
C8—C9	1.438 (3)	C23—C24	1.394 (4)
C8—H8A	0.9500	C23—H23A	0.9500
C9—C12	1.417 (3)	C24—C25	1.389 (3)
C9—C10	1.427 (3)	C24—H24A	0.9500
C10—C15	1.410 (3)	C25—H25A	0.9500
C10—C11	1.460 (3)		
			
C11—N1—N2	113.26 (18)	C12—C13—H13A	119.9
C11—N1—C7	125.3 (2)	C14—C13—H13A	119.9
N2—N1—C7	121.39 (18)	C15—C14—C13	120.4 (2)
C17—N2—N1	103.16 (19)	C15—C14—H14A	119.8
C18—O2—C19	116.05 (19)	C13—C14—H14A	119.8
C2—C1—C6	120.3 (2)	C14—C15—C10	120.7 (2)
C2—C1—H1A	119.8	C14—C15—H15A	119.7
C6—C1—H1A	119.8	C10—C15—H15A	119.7
C3—C2—C1	120.2 (2)	C17—C16—C11	105.01 (19)
C3—C2—H2B	119.9	C17—C16—C18	124.5 (2)
C1—C2—H2B	119.9	C11—C16—C18	130.4 (2)
C2—C3—C4	119.9 (2)	N2—C17—C16	113.8 (2)
C2—C3—H3A	120.0	N2—C17—H17A	123.1
C4—C3—H3A	120.0	C16—C17—H17A	123.1
C3—C4—C5	120.3 (2)	O1—C18—O2	122.1 (2)
C3—C4—H4A	119.8	O1—C18—C16	128.4 (2)
C5—C4—H4A	119.8	O2—C18—C16	109.6 (2)
C4—C5—C6	119.0 (2)	O2—C19—C20	111.82 (18)
C4—C5—C7	119.0 (2)	O2—C19—H19A	109.3
C6—C5—C7	121.9 (2)	C20—C19—H19A	109.3
C1—C6—C5	120.2 (2)	O2—C19—H19B	109.3
C1—C6—H6A	119.9	C20—C19—H19B	109.3
C5—C6—H6A	119.9	H19A—C19—H19B	107.9
C8—C7—N1	117.0 (2)	C21—C20—C25	117.8 (2)
C8—C7—C5	123.4 (2)	C21—C20—C19	119.9 (2)
N1—C7—C5	119.6 (2)	C25—C20—C19	122.3 (2)
C7—C8—C9	122.3 (2)	C22—C21—C20	121.0 (3)
C7—C8—H8A	118.9	C22—C21—H21A	119.5
C9—C8—H8A	118.9	C20—C21—H21A	119.5
C12—C9—C10	118.8 (2)	C23—C22—C21	120.3 (3)
C12—C9—C8	121.1 (2)	C23—C22—H22A	119.9
C10—C9—C8	120.1 (2)	C21—C22—H22A	119.9
C15—C10—C9	119.0 (2)	C22—C23—C24	119.9 (3)
C15—C10—C11	123.4 (2)	C22—C23—H23A	120.1
C9—C10—C11	117.6 (2)	C24—C23—H23A	120.1
N1—C11—C16	104.8 (2)	C25—C24—C23	119.7 (3)
N1—C11—C10	117.6 (2)	C25—C24—H24A	120.1
C16—C11—C10	137.6 (2)	C23—C24—H24A	120.1
C13—C12—C9	121.0 (2)	C24—C25—C20	121.4 (3)
C13—C12—H12A	119.5	C24—C25—H25A	119.3
C9—C12—H12A	119.5	C20—C25—H25A	119.3
C12—C13—C14	120.1 (2)		
			
C11—N1—N2—C17	0.4 (2)	C9—C10—C11—C16	178.3 (2)
C7—N1—N2—C17	177.82 (19)	C10—C9—C12—C13	1.7 (4)
C6—C1—C2—C3	0.5 (4)	C8—C9—C12—C13	−179.1 (2)
C1—C2—C3—C4	0.2 (4)	C9—C12—C13—C14	0.7 (4)
C2—C3—C4—C5	−1.0 (4)	C12—C13—C14—C15	−2.5 (4)
C3—C4—C5—C6	1.0 (4)	C13—C14—C15—C10	1.9 (4)
C3—C4—C5—C7	−174.8 (2)	C9—C10—C15—C14	0.5 (3)
C2—C1—C6—C5	−0.5 (4)	C11—C10—C15—C14	−179.2 (2)
C4—C5—C6—C1	−0.3 (4)	N1—C11—C16—C17	0.8 (2)
C7—C5—C6—C1	175.4 (2)	C10—C11—C16—C17	−176.7 (2)
C11—N1—C7—C8	−0.3 (3)	N1—C11—C16—C18	−177.4 (2)
N2—N1—C7—C8	−177.46 (19)	C10—C11—C16—C18	5.1 (4)
C11—N1—C7—C5	−179.4 (2)	N1—N2—C17—C16	0.2 (2)
N2—N1—C7—C5	3.4 (3)	C11—C16—C17—N2	−0.7 (3)
C4—C5—C7—C8	54.6 (3)	C18—C16—C17—N2	177.6 (2)
C6—C5—C7—C8	−121.1 (3)	C19—O2—C18—O1	0.0 (3)
C4—C5—C7—N1	−126.4 (2)	C19—O2—C18—C16	−179.81 (17)
C6—C5—C7—N1	58.0 (3)	C17—C16—C18—O1	−171.4 (2)
N1—C7—C8—C9	−0.4 (3)	C11—C16—C18—O1	6.5 (4)
C5—C7—C8—C9	178.6 (2)	C17—C16—C18—O2	8.4 (3)
C7—C8—C9—C12	−177.7 (2)	C11—C16—C18—O2	−173.7 (2)
C7—C8—C9—C10	1.5 (4)	C18—O2—C19—C20	−96.2 (2)
C12—C9—C10—C15	−2.2 (3)	O2—C19—C20—C21	−137.2 (2)
C8—C9—C10—C15	178.5 (2)	O2—C19—C20—C25	43.3 (3)
C12—C9—C10—C11	177.5 (2)	C25—C20—C21—C22	1.5 (3)
C8—C9—C10—C11	−1.7 (3)	C19—C20—C21—C22	−178.0 (2)
N2—N1—C11—C16	−0.8 (2)	C20—C21—C22—C23	−1.2 (4)
C7—N1—C11—C16	−178.12 (19)	C21—C22—C23—C24	0.5 (4)
N2—N1—C11—C10	177.36 (18)	C22—C23—C24—C25	−0.1 (4)
C7—N1—C11—C10	0.0 (3)	C23—C24—C25—C20	0.5 (4)
C15—C10—C11—N1	−179.3 (2)	C21—C20—C25—C24	−1.2 (3)
C9—C10—C11—N1	1.0 (3)	C19—C20—C25—C24	178.4 (2)
C15—C10—C11—C16	−1.9 (4)		

### Hydrogen-bond geometry (Å, °)

**Table:**

Cg1 and Cg2 are the centroids of the N1/N2C11/C16/C17 and C20–C25 rings, respectively.

**Table:**

*D*—H···*A*	*D*—H	H···*A*	*D*···*A*	*D*—H···*A*
C15—H15A···O1	0.95	2.17	3.012 (3)	148
C1—H1A···Cg1^i^	0.95	2.76	3.484 (3)	134
C14—H14A···Cg2^ii^	0.95	2.68	3.594 (3)	161

Symmetry codes: (i) *x*, −*y*+1/2, *z*−1/2; (ii) *x*+1, *y*, *z*.
